# Olanzapine Treatment of Adolescent Rats Alters Adult D2 Modulation of Cortical Inputs to the Ventral Striatum

**DOI:** 10.1093/ijnp/pyw034

**Published:** 2016-04-27

**Authors:** Julie M. Brooks, Patricio O’Donnell, Douglas O. Frost

**Affiliations:** Department of Anatomy and Neurobiology (Drs Brooks and O’Donnell), Department of Psychiatry (Drs O’Donnell and Frost), and Department of Pharmacology (Dr Frost), University of Maryland School of Medicine, University of Maryland, Baltimore, Maryland.

**Keywords:** antipsychotic drugs, olanzapine, rat, adolescence, ventral striatum

## Abstract

**Background:**

The striatal dopamine system undergoes vast ontogenetic changes during adolescence, making the brain vulnerable to drug treatments that target this class of neurotransmitters. Atypical antipsychotic drugs are often prescribed to children and adolescents for off-label treatment of neuropsychiatric disorders, yet the long-term impact this treatment has on brain development remains largely unknown.

**Methods:**

Adolescent male rats were treated with olanzapine or vehicle for 3 weeks (during postnatal day 28–49) using a dosing condition designed to approximate closely D2 receptor occupancies in the human therapeutic range. We assessed D2 receptor modulation of corticostriatal inputs onto medium spiny neurons in the adult ventral striatum using in vitro whole-cell current clamp recordings.

**Results:**

The D2/D3 agonist quinpirole (5 µM) enhanced cortically driven medium spiny neuron synaptic responses in slices taken from adult rats treated with vehicle during adolescence, as in untreated adult rats. However, in slices from mature rats treated with olanzapine during adolescence, quinpirole reduced medium spiny neuron activation. The magnitude of decrease was similar to previous observations in untreated, prepubertal rats. These changes may reflect alterations in local inhibitory circuitry, as the GABA-A antagonist picrotoxin (100 µM) reversed the effects of quinpirole in vehicle-treated slices but had no impact on cortically evoked responses in olanzapine-treated slices.

**Conclusions:**

These data suggest that adolescent atypical antipsychotic drug treatment leads to enduring changes in dopamine modulation of corticostriatal synaptic function.

## Introduction

Adolescence is a critical developmental stage during which corticostriatal circuits and their modulation by dopamine (DA) are still maturing. The protracted developmental trajectory of dopaminergic projections in rodents (Tseng and O’Donnell, 2007; Benoit-Marand and O’Donnell, 2008; Huppe-Gourgues and O’Donnell, 2012) and humans (Casey et al., 2010; Galvan, 2010) makes these circuits especially vulnerable to the effects of psychotropic drug treatment during adolescence. Second-generation atypical antipsychotic drugs (AAPDs), such as olanzapine (OLA), are commonly prescribed for adolescent psychosis (Baeza et al., 2014) and are often used off-label for a variety of other neuropsychiatric and behavioral indications (Domino and Swartz, 2008; Olfson et al., 2010). Although studies of adolescent antipsychotic treatment have increased over recent years, the primary focus of this research has been on drug efficacy and tolerance. While even time-limited AAPD therapy can alter brain developmental trajectories, the long-term consequences of AAPD treatment of children and adolescents remain largely unknown.

Recent work using animal models has begun to shed light on these issues. AAPDs administered to adolescent rodents at doses that produce D2 receptor occupancies in the human therapeutic range result in a variety of long-term behavioral and neurobiological effects. We (Milstein et al., 2013; Vinish et al., 2013) and others (Llorente-Berzal et al., 2012) have shown that adult rats treated with OLA at these doses during adolescence exhibit abnormalities in learning tasks that are sensitive to DA function. These rats also exhibit changes in DA transmission (Milstein et al., 2013; Vinish et al., 2013), glutamate (GLU), and GABA levels (Xu et al., 2015) and the organization of neuronal networks (Milstein et al., 2013) in several brain regions that are mediators of the affected behaviors, including the ventral striatum (VS). The VS, which includes the nucleus accumbens (NAc) and the medial aspect of the dorsal striatum, is defined by its connectivity with limbic regions, including the medial prefrontal cortex, ventral hippocampus, and amygdala (Voorn et al., 2004). DA transmission in the VS is believed to fine tune the dynamic integration of the diverse GLU inputs that converge upon this region (O’Donnell and Grace, 1994; O’Donnell, 2003; Brady and O’Donnell, 2004), and the nature of this modulation changes dramatically during adolescence. In prepubertal rats, D2 receptor activation attenuates excitatory postsynaptic potentials (EPSPs) evoked in NAc medium spiny neurons (MSNs) by stimulation of corticostriatal afferents. Instead, D2 activation in adult rats potentiates cortically evoked EPSPs, and this response involves recruitment of local GABAergic transmission (Benoit-Marand and O’Donnell, 2008). We also reported peripubertal changes in D2 receptor modulation of AMPA-driven MSN excitability in the VS (Huppe-Gourgues and O’Donnell, 2012). These data provided evidence of a maturational change in the activation of local GABA circuitry by D2 family receptors. Here, we evaluated the long-term impact of adolescent OLA treatment on D2 receptor modulation of GLU cortical afferents to the VS with whole-cell recordings of corticostriatal synaptic responses in MSNs of the VS of adult rats treated as adolescents with OLA or vehicle (VEH).

## Methods

### Subjects

Adult (94–181 days old), male Long-Evans rats (350–450g), colony-bred (breeding stock from Charles River Laboratory: Wilmington, MA) and treated during adolescence, served as subjects for all experiments. On postnatal day (PD) 7, litters were culled to 10 to 12 pups. On PD21, rats were weaned and pair- or triple-housed with same-sex littermates. The rats were maintained in a temperature- and humidity-controlled environment on a 12-hour-light/-dark cycle (lights on at 6:30 am) with food and water available ad libitum. A subset of rats was initially housed in a reversed light cycle (lights on at 6:30 pm) but were given 2 weeks to habituate to the regular light cycle prior to testing. Animal care and experimentation were performed in accordance with protocols approved by The University of Maryland School of Medicine Institutional Animal Care and Use Committee and consistent with the NIH Guide for the Care and Use of Laboratory Animals.

### Adolescent Drug Treatment

Our drug administration protocols were previously described (Milstein et al., 2013; Vinish et al., 2013). Briefly, on PD28 to 49, drinking water was replaced with an aqueous solution of OLA in 1mM acetic acid or VEH. Each day the OLA solution was mixed fresh at a concentration calculated to deliver a target dose of 7.5mg/kg/d based on the weight and water consumption of the rats during the previous 24 hours. This regimen achieved an average of approximately 96% of the target dose. All subjects were switched to normal drinking water on PD50. This protocol was designed to approximate closely D2 receptor occupancies in the human therapeutic range (Kapur et al., 2003).

### Electrophysiology

Rats were anesthetized with chloral hydrate (400mg/kg, i.p.) and transcardially perfused prior to decapitation with oxygenated ice-cold artificial cerebral spinal fluid (aCSF) containing (in mM): NaCl, 125; NaHCO_3_, 25; glucose, 10; KCl, 3.5mM; NaH_2_PO_4_, 1.25; CaCl_2_, 0.5mM; MgCl_2_, 3mM; pH 7.4, osmolarity 295 mOsm, constantly oxygenated with 95% O_2_ and 5% CO_2_. Parasagittal slices (300 µm thick) cut at a 10^o^ angle containing the VS and corticostriatal fibers were sectioned using a Vibratome. Slices were incubated in oxygenated aCSF warmed to approximately 34^o^C for at least 1 hour prior to recording. For each experiment, slices were placed in a submersion-type recording chamber superfused with oxygenated aCSF at a flow rate of 2mL/min and maintained at 33^o^C to 34^o^C. Recording aCSF formulations were adjusted to include 2mM CaCl_2_ and 1mM MgCl_2_.

Whole-cell current clamp recordings were performed from MSNs within the VS of brain slices obtained from adult rats treated as adolescents with either OLA or VEH. Ventral striatal MSNs were identified using infrared differential interference contrast video microscopy (Olympus BX50-WI) using a 40x water-immersion objective. Visual guidance was obtained with an IR-sensitive CCD camera (DAGE-MTI) connected to a monitor. Patch pipettes (6–10 MΩ) were made from 1.5-mm O.D. borosilicate glass tubing (World Precision Instruments, Sarasota, FL) and filled with (in mM): K-gluconate, 115; HEPES, 10; MgCl_2_ 2; KCl, 20; Mg-ATP, 2; Na_2_-ATP, 2; and GTP, 0.3 (pH 7.3; osmolarity 280 mOsm). Neurobiotin (0.125%) was added to the internal recording solution for histological identification of recorded cells. Whole-cell recordings were acquired with a computer-controlled Multiclamp 700B amplifier (Axon Instruments, Foster City, CA), digitized (Digidata, Axon Instruments), and sampled with an Axoscope 9.0 (Axon Instruments) at a rate of 10kHz. Electrode potentials were adjusted to zero before recording without correcting the liquid junction potential.

**Figure 1.**
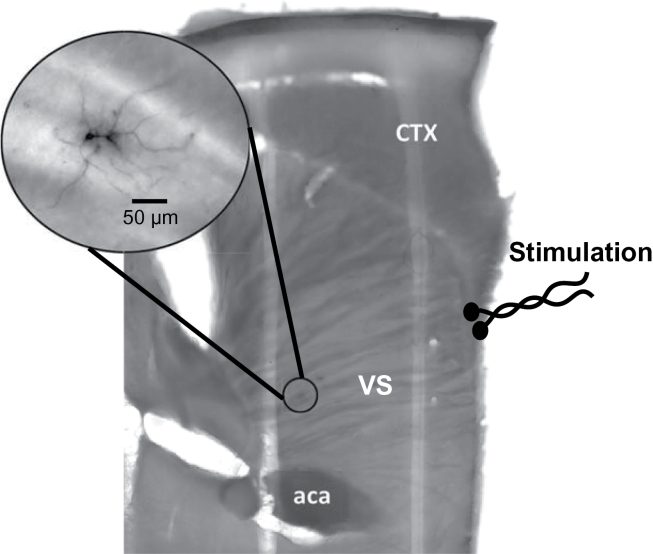
Photomicrograph of a representative parasagittal brain slice illustrating the placement of the stimulating electrode with respect to the recording region. Inset: medium spiny neurons (MSNs) filled with neurobiotin during the recording session and stained using 3,3’ –diaminobenzidine tetrachloride. aca, anterior commissure; CTX, cortex; VS, ventral striatum.

Synaptic responses were evoked in VS MSNs via electrical stimulation of corticostriatal fiber tracts (0.2–0.9 mA, 0.5-ms duration) every 15 seconds using a computer programed Master 8 pulse generator (A.M.P.I., Jerusalem, Israel). EPSPs were evoked using a bipolar electrode made from a twisted pair of Teflon-coated tungsten wires (tips approximately 200 µm apart). The stimulating electrode was placed in the forceps minor, approximately 500 µm from the recorded cell (Figure 1). The initial 5min after the seal was broken was used to stabilize recordings. This was followed by a full assessment of passive membrane properties, including membrane potential and input resistance (measured using the slope of a current-voltage plot obtained with 500-ms hyperpolarizing and depolarizing pulses) before and after bath application of drugs. Neurons exhibiting a resting membrane potential more depolarized than -70 mV and/or an input resistance <80 MΩ were excluded from analysis. Once a steady baseline was established, activity was recorded for 10 minutes prior to drug application. Solutions containing drugs were superfused for 5 minutes followed by aCSF superfusion. Baseline values for all parameters were calculated by averaging EPSPs over a 2-minute period directly preceding drug application. Similarly, drug effects were calculated by averaging EPSP values during a 2-minute period starting 3 minutes after the onset of drug application to allow the stabilization of drug levels within the recording area. Drugs utilized include the D2 family agonist (-)-quinpirole, the GABA-A antagonist picrotoxin, and the competitive AMPA/kainate receptor blocker 6-cyano-7-nitroquinoxaline-2,3-dione. All drugs were purchased from Sigma (St. Louis, MO).

### Histological Procedures

At the end of each experiment, all slices were placed in 4% paraformaldehyde overnight. Slices were then washed in 0.1M phosphate buffered saline and incubated with 0.3% Triton X-100 in phosphate buffered saline. To block endogenous peroxidases, slices were incubated for 15min in 0.3% hydrogen peroxide. The avidin-biotin complex method was used to detect Neurobiotin-filled cells (ABC peroxidase kit; Vector Laboratories, Burlingame, CA), and the reaction was visualized using 3,3’ –diaminobenzidine tetrachloride (Sigma) to provide verification of the morphology and location of recorded neurons (Figure 1).

### Statistical Analyses

Data are expressed as mean±SD. For each experimental measure, the main effects of adolescent treatment and drug application to the bath were assessed using repeated-measures ANOVA. When appropriate, a minimum number of posthoc comparisons was conducted using a Wilcoxon or a Mann-Whitney test (for paired and unpaired observations, respectively) implemented in SPSS for Windows (V15.0; SPSS Inc. Chicago, IL).

## Results

### Passive Membrane Properties of Adult VS MSNs Are Unaffected by Adolescent OLA Treatment

Fifty-two MSNs were recorded in VS slices obtained from adult rats treated as adolescents with either OLA (n=25) or VEH (n=27). Only those cells verified as MSNs by their soma-dendritic form and neurobiotin immunopositivity (Figure 1) were included in our analyses. Animals from each treatment group were drawn from ≥3 litters, and each litter included animals in both treatment groups to minimize uncontrolled litter effects. There were no significant group differences in resting membrane potential (-75.0±2.6 mV in VEH-treated rats and –74.4±2.3 mV in OLA-treated rats; *P*=.52). Input resistances were also similar across treatment conditions (117.1±24.0 MΩ in VEH-treated rats and 124.8±26.2 MΩ in OLA-treated rats; *P*=.29). Thus, adolescent OLA treatment did not impact basic membrane properties of mature MSNs in the VS.

### Adolescent OLA Treatment Alters Adult D2 Modulation of Corticostriatal Synaptic Responses

**Figure 2.**
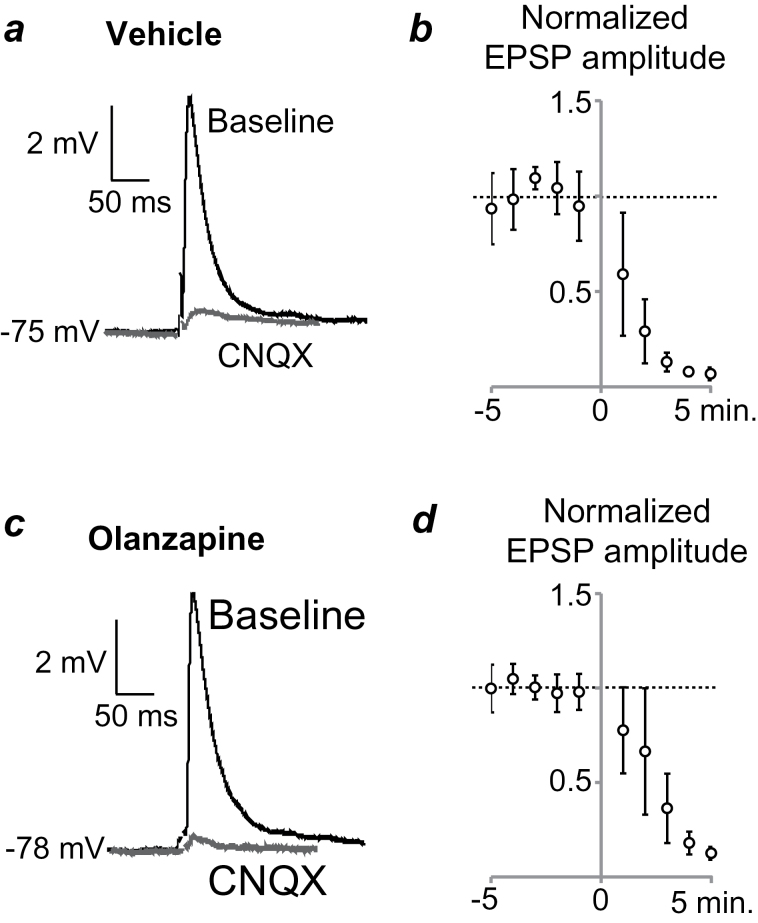
AMPA/kainate receptor blockade inhibits responsiveness of ventral striatal medium spiny neurons (MSNs) to corticostriatal afferents. (*a*) Responses to electrical stimulation of corticostriatal fiber tracts before (baseline) and during bath application of 6-cyano-7-nitroquinoxaline-2,3-dione (CNQX) (10 µM) in a representative MSN from an adult rat treated with vehicle (VEH) during adolescence. Each trace is an average of 10 sweeps. (*b*) Group data on excitatory postsynaptic potential (EPSP) amplitudes averaged over 1 minute and normalized to the mean amplitude prior to CNQX application. CNQX administration at time zero causes a progressive loss of responsiveness in MSNs from VEH-treated rats. (*c*) Averaged EPSPs in a representative MSN from an adult rat treated with olanzapine (OLA) as an adolescent. (*d*) Group data of normalized EPSP amplitudes in MSNs from OLA-treated rats. As in VEH-treated control rats, there is a progressive loss of responsiveness following CNQX administration.

**Figure 3.**
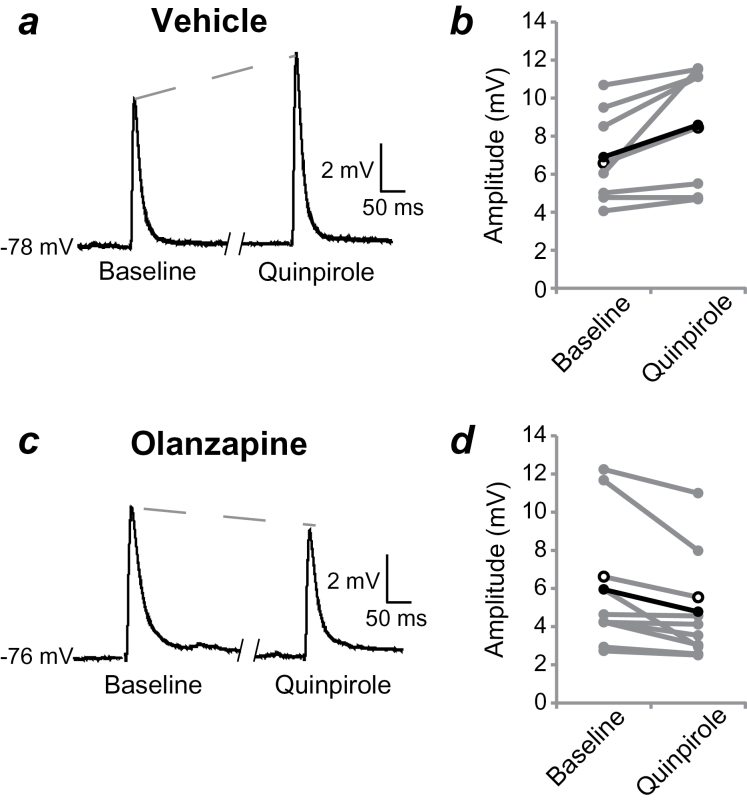
Adolescent olanzapine (OLA) treatment alters D2 receptor modulation of medium spiny neuron (MSN) responses to cortical afferent stimulation. (*a*) Responses before (baseline) and during bath application of quinpirole (5 µM) in a representative MSN from an adult rat treated with vehicle (VEH) as an adolescent. Each trace is the average of 10 sweeps. (*b*) Averaged excitatory postsynaptic potential (EPSP) amplitudes for individual MSNs from VEH-treated rats are shown in grey. Quinpirole administration increases EPSP amplitude (in this and subsequent figures, data from the cell whose averaged traces are illustrated are indicated by open black circles; group averages are indicated by closed black circles). (*c*) Averaged EPSPs in a representative MSN from an adult rat treated with olanzapine (OLA) as an adolescent. (*d*) Averaged EPSP amplitudes for individual MSNs from OLA-treated rats. Quinpirole administration decreases EPSP amplitude in adult MSN when the rats had been treated with OLA during adolescence.

Electrical stimulation of the forceps minor, the white matter giving rise to corticostriatal fibers, consistently evoked EPSPs in VS MSNs located 0.5 to 1mm away from the stimulation site in rats of both treatment groups. Bath application of the AMPA receptor antagonist cyano-7-nitroquinoxaline-2,3-dione (10 µM) nearly abolished the evoked responses, confirming their glutamatergic nature (Figure 2). To investigate the long-term impact of adolescent OLA treatment on D2 modulation of corticostriatal synaptic activity, we assessed EPSP amplitudes before and after bath application of quinpirole (5 μM). An initial comparison revealed that D2 receptor modulation of corticostriatal synaptic responses differed between VEH- and OLA-treated rats (F_1,16_=16.438, *P*=.001). In adult VEH-treated rats, quinpirole administration significantly increased EPSP amplitude by approximately 25% (from 6.9±2.3 mV to 8.6±3.2 mV; *P*=.018, n=8) (Figure 3*a-b*), consistent with previous observations in MSNs of untreated adult rats (Benoit-Marand and O’Donnell, 2008). In contrast, quinpirole administration significantly decreased EPSP amplitudes by approximately 18% (from 6.0±3.2 mV to 4.8±2.8 mV; *P*=.005, n=10) (Figure 3*c-d*) in MSNs of adult rats treated with OLA during adolescence. This decrease in synaptic response is similar to that obtained previously in MSNs of untreated, prepubertal rats (PD23-38) (Benoit-Marand and O’Donnell, 2008). These data suggest that D2 receptor antagonism during the maturation of the mesolimbic pathway causes long-term changes in DA modulation of corticostriatal synaptic activity, resulting in a prolonged adolescent profile of MSN responses to cortical afferent activation.

### Adolescent OLA Treatment-Evoked Alterations in D2 Modulation of Corticostriatal Activity Include a GABAergic Component

**Figure 4.**
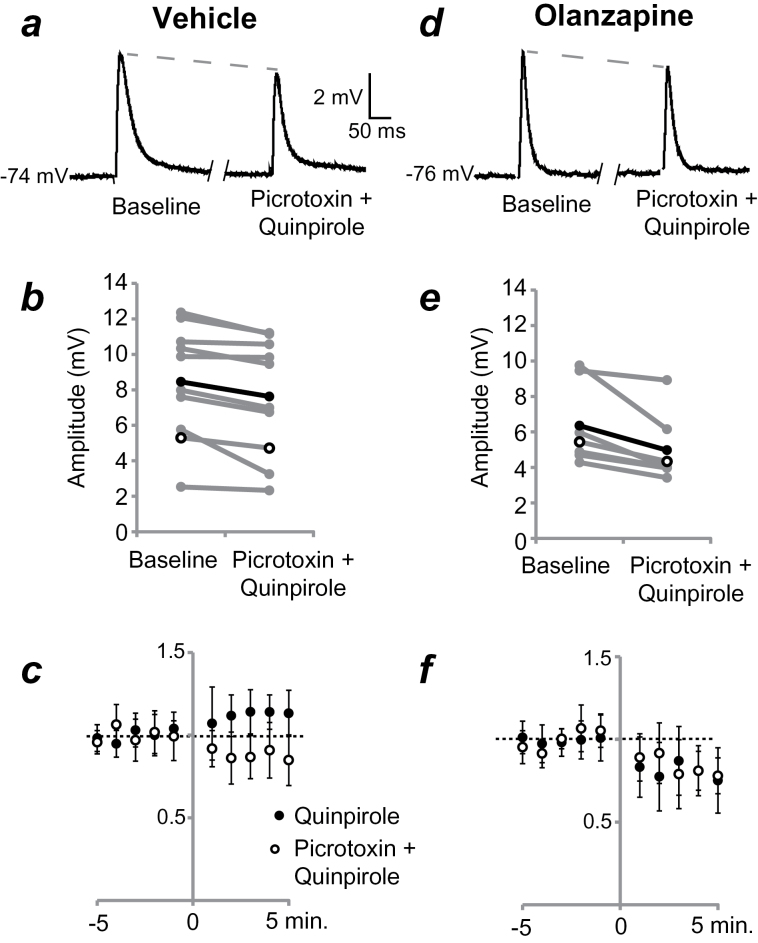
Changes in the D2 receptor mediated modulation of medium spiny neuron (MSN) responses to stimulation of corticostriatal afferents involve a GABA-A component. (*a*) Responses before (baseline) and during coadministration of the GABA-A receptor antagonist picrotoxin (100 µM) and quinpirole in a representative MSN from an adult rat treated with vehicle (VEH) as an adolescent (traces are averages of 10 sweeps). (*b*) Averaged excitatory postsynaptic potential (EPSP) amplitudes for individual MSNs from VEH-treated rats. Picrotoxin coadministered with quinpirole decreases EPSP amplitudes in the VEH-treated group. (*c*) Group data on EPSP amplitudes (averaged over 1 minute and normalized, as in Figure 2) highlight the change in D2 receptor modulation of EPSP amplitude caused by GABA-A blockade in VEH-treated rats (between-subjects comparison). (*d*) Averaged traces from a representative MSN from an adult rat treated with olanzapine (OLA) as an adolescent. (*e*) Averaged EPSP amplitudes for individual MSNs from OLA-treated rats. Picrotoxin coadministered with quinpirole decreases responses to stimulation of corticostriatal afferents. (*f*) Normalized group data from OLA-treated rats. GABA-A receptor blockade does not significantly alter the quinpirole-induced decrease in EPSP amplitude (compared between subjects).

**Figure 5.**
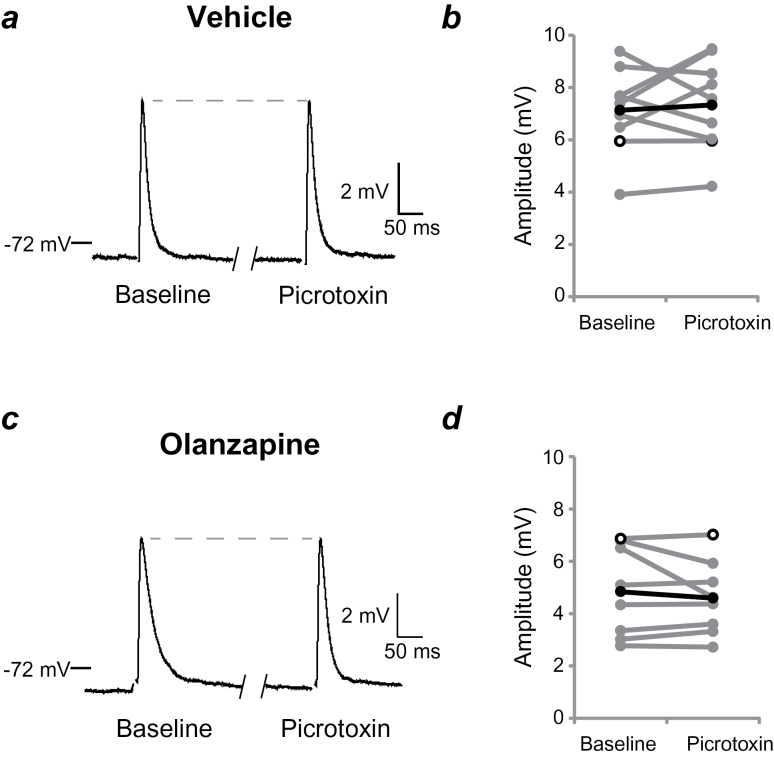
GABA-A receptor blockade alone does not significantly alter medium spiny neuron (MSN) responses to cortical afferent stimulation in adult rats. (*a*) Responses before (baseline) and during bath application of picrotoxin (100 µM) in a representative MSN from an adult rat treated with vehicle (VEH) as an adolescent. Each trace is the average of 10 sweeps. (*b*) Group data on averaged excitatory postsynaptic potential (EPSP) amplitudes for individual MSNs from VEH-treated rats. (*c*) Representative traces and (*d*) group data from adult rats treated with olanzapine (OLA) during adolescence.

Previous data from our laboratory indicate that developmental changes in D2 family receptor modulation of corticostriatal synaptic responses in the VS are attributable to the recruitment of a local depolarizing GABAergic mechanism that emerges during adolescence (Benoit-Marand and O’Donnell, 2008). It is possible OLA treatment during this critical period of development inhibits that process, yielding adult MSNs that retain a D2-mediated attenuation of corticostriatal synaptic responses but lack the added modulation by local GABAergic recruitment. To test this hypothesis, we repeated our experiments in the presence of the GABA-A receptor antagonist picrotoxin (100 μM) in a second cohort of rats. As in slices obtained from untreated, adult rats (Benoit-Marand and O’Donnell, 2008), coadministration of picrotoxin with quinpirole (5 μM) significantly decreased EPSP amplitude by approximately 12% (from 8.5±3.0 mV to 7.6±3.2 mV; *P*=.005; n=10) (Figure 4*a-b*) in MSNs of adult rats treated with VEH during adolescence. This observation contrasts with the increase observed in normal rats (Benoit-Marand and O’Donnell, 2008) and the VEH-treated group in the absence of GABA-A antagonism (Figures 3*a*, 4*c*). Conversely, GABA-A receptor blockade did not alter the D2-mediated attenuation of cortically evoked EPSPs in MSNs from adult rats treated with OLA as adolescents (*P*=.30; n=7) (Figure 4*d-f*). Picrotoxin alone did not have a significant effect on EPSP amplitude in either treatment condition (VEH n=9, OLA n=8, *P*=.69) (Figure 5). As indicated above, input resistance was not affected by picrotoxin in either treatment condition. Furthermore, quinpirole or quinpirole plus picrotoxin did not alter input resistance (VEH treated: 115±14 MΩ to 121±24 MΩ with quinpirole, 121±18 MΩ to 122±24 MΩ with quinpirole and picrotoxin; OLA treated: 118±22 MΩ to 130.6 MΩ ± 34 with quinpirole, 117±24 MΩ to 123±22 MΩ with quinpirole and picrotoxin). The lack of changes in input resistance suggests the effects observed are likely due to presynaptic effects and not direct effects on the recorded neurons. These results further substantiate the involvement of D2 receptors in the recruitment of local inhibitory mechanisms during corticostriatal activation of MSNs in normal adults and suggest that adolescent exposure to AAPDs could alter D2 receptor recruitment of this neuron subpopulation.

## Discussion

These experiments demonstrate that OLA treatment during adolescence alters adult D2 receptor modulation of corticostriatal responses. Quinpirole administration significantly enhanced synaptic responses in slices obtained from adult rats treated with VEH on PD28 to 49 but attenuated MSN activation in mature rats treated with OLA during the same epoch. The magnitude of suppression observed in adult OLA-treated rats was similar to that in normal prepubertal rats (Benoit-Marand and O’Donnell, 2008). In the presence of the GABA-A antagonist, picrotoxin, D2 receptor activation attenuated cortically evoked EPSPs in VEH-treated adult rats, as it does in untreated control rats both pre- and postpubertally. In OLA-treated adult rats, the combination of picrotoxin and quinpirole decreased EPSP magnitude, as for D2 receptor activation with quinpirole alone. These data suggest that adolescent OLA treatment alters the developmental trajectory of DA modulation of corticostriatal synaptic function in the VS.

Our data show that D2 receptor antagonism by OLA during this period disrupts the developmental trajectory of DA modulation of corticostriatal responses in MSNs. As no differences in basic membrane properties were observed between MSNs of adult rats treated with OLA and VEH during adolescence, it is unlikely that the impact is on MSN physiology. Indeed, recorded cells from both adolescent treatment groups exhibited resting membrane potentials between -70 mV and -80 mV and input resistances between 93 and 151 MΩ, values similar to those previously described in untreated adult rats (O’Donnell and Grace, 1993). Furthermore, quinpirole alone has no effect on MSN membrane potential or input resistance (O’Donnell and Grace, 1994, 1996; Huppe-Gourgues and O’Donnell, 2012). Rather, the changes observed likely reflect altered D2 modulation of inputs to MSNs.

The modulation of corticostriatal synaptic responses by D2 receptors changes dramatically around adolescence (Benoit-Marand and O’Donnell, 2008; Huppe-Gourgues and O’Donnell, 2012). Quinpirole decreased the amplitude of EPSPs evoked in MSNs by stimulation of cortical afferents in adult rats (>PD90) treated with OLA during adolescence. This reduction normally occurs in prepubertal, but not adult, rats. These data suggest that D2 receptor antagonism during the maturation of the mesolimbic pathway leads to a prolonged adolescent profile of MSN responses to cortical afferent activation. In the adult rat, D2 receptor activation is thought to enhance MSN synaptic responses to cortical stimulation by engaging fast-spiking, parvalbumin immunoreactive, GABAergic interneurons (Benoit-Marand and O’Donnell, 2008). This is evidenced by the emergence of GLU-independent inward currents in MSNs following quinpirole administration, an effect reversed only by GABA-A receptor blockade (Benoit-Marand et al., 2008). Recruitment of this neuronal population can have a depolarizing effect in target neurons with resting membrane potentials below the K^+^ equilibrium potential (Koos and Tepper, 1999). Parvalbumin positive interneurons can be activated by corticostriatal inputs (Mallet et al., 2005; Gruber et al., 2009a) and modulate responses in VS to diverse inputs (Calhoon and O’Donnell, 2013). This feed-forward mechanism is intact in VEH-treated control animals, as quinpirole administration enhanced corticostriatal EPSPs and the effect was reversed by the administration of the GABA-A open channel blocker picrotoxin. However, D2 receptor recruitment of depolarizing GABAergic responses was absent in OLA-treated rats as evidenced by quinpirole decreasing, rather than -enhancing, EPSP amplitude in MSNs and the persistence of this effect in the presence of picrotoxin. This possibility is consistent with the reduced levels of GABA in the NAc of adult rats treated with OLA as adolescents (Xu et al., 2015). OLA treatment could reduce D2 receptor binding on interneurons or alter the coupling of those receptors to G-proteins. Alternatively, it could increase D2 receptor binding on presynaptic corticostriatal axon terminals that synapse on the interneurons, thus reducing glutamatergic transmission at those synapses. This hypothesis is consistent with the reduction of GLU levels (Xu et al., 2015) and the overall increase in D2 receptor binding (Vinish et al., 2013) in the NAc of adult rats treated with OLA as adolescents. Thus, D2 receptor-mediated effects on MSN activation are likely to involve local interneuron activation, and adolescent OLA treatment may block the emergence of this mechanism. The result is a striatal circuit in which D2 direct modulation of MSN is not balanced by interneuron recruitment, and the net effect of DA via D2 receptors is to attenuate corticostriatal transmission.

The effect of adolescent OLA treatment on the modulation of corticostriatal synaptic responses by DA reported here is likely to drive a number of behavioral changes. Indeed, adult rats treated with OLA during adolescence exhibit deficits in several behavioral paradigms associated with corticostriatal afferent activation, including working memory and conditioned place preference (Milstein et al., 2013; Vinish et al., 2013). Furthermore, these behavioral changes are accompanied by reductions in DA release and in GABA and GLU levels in the NAc, and disrupted DA and GABA signaling in the PFC (Milstein et al., 2013; Vinish et al., 2013; Xu et al., 2015). Abnormal behavioral outcomes in OLA-treated rats were obtained at 3 to 8.5 months of age (Milstein et al., 2013; Vinish et al., 2013), an age range similar to that showing the electrophysiological changes we report here. The present and previous studies (Llorente-Berzal et al., 2012; Milstein et al., 2013; Vinish et al., 2013; Xu et al., 2015) show that adolescent OLA treatment induces a variety of enduring behavioral and neurobiological effects. Our data suggest that D2 receptor antagonism by adolescent OLA treatment has long-lasting consequences that affect the normal development of corticoaccumbens synapses, specifically their modulation by DA.

The neurobiological impact of antipsychotic drug therapy can vary depending on the homeostatic effect of the treatment, which can change over the protracted course of brain development. The current study focused specifically on the enduring changes that occur following antipsychotic treatment during adolescence, a period of dynamic change in the DA system. This is not to say that alterations in D2 modulation of corticostriatal synaptic physiology would not occur in rats treated as adults following a similar treatment protocol and posttreatment interval. While we are unaware of any data addressing this exact question, numerous studies have demonstrated that chronic AAPD treatment in adult rats significantly impacts striatal DA, GLU, and GABA neurotransmission (Lieberman et al., 2008). However, those data provide no insight as to the effects of D2 receptor antagonism on substrates particularly vulnerable to DA modulation, as would be the case during adolescence. We also emphasize that the effects of adolescent OLA treatment reported here and in other studies are present long after the cessation of a brief, subchronic (3-week) exposure (Milstein et al., 2013; Vinish et al., 2013; Xu et al., 2015). The enduring effects of APD therapy following time-limited treatment of adults have not, to our knowledge, been systematically investigated.

Corticostriatal interactions are critical for decision making and reward-based learning (Christakou et al., 2004; Cohen et al., 2009). The VS integrates input from multiple afferent pathways, including the prefrontal cortex, hippocampus, and amygdala (Brady and O’Donnell, 2004; Gruber et al., 2009b; Calhoon and O’Donnell, 2013), which convey emotional and contextual information. DA and GABA modulation of this process is critical for the normal function of the VS at maturity. Our data predict that modulation of DA function by adolescent AAPD therapy in humans carries a strong risk of impaired decision-making and reward processing at maturity. Thus, AAPD treatment of adolescents should be used only when absolutely necessary. This consideration underscores the importance of developing new therapeutic strategies that would mitigate these adverse effects.

## Statement of Interest

Patricio O’Donnell is an employee and shareholder of Pfizer, Inc. All work reported here was conducted prior to Dr. O’Donnell joining Pfizer.
